# Comprehensive Profiling of Genomic and Transcriptomic Differences between Risk Groups of Lung Adenocarcinoma and Lung Squamous Cell Carcinoma

**DOI:** 10.3390/jpm11020154

**Published:** 2021-02-23

**Authors:** Talip Zengin, Tuğba Önal-Süzek

**Affiliations:** 1 Department of Molecular Biology and Genetics, Muğla Sıtkı Koçman University, 48000 Muğla, Turkey; talipzengin@mu.edu.tr; 2 Department of Bioinformatics, Muğla Sıtkı Koçman University, 48000 Muğla, Turkey; 3 Department of Computer Engineering, Muğla Sıtkı Koçman University, 48000 Muğla, Turkey

**Keywords:** TCGA, non-small-cell lung cancer, lung adenocarcinoma (LUAD), lung squamous cell carcinoma (LUSC), differential expression, SNV, CNV, risk group, signature, survival

## Abstract

Lung cancer is the second most frequently diagnosed cancer type and responsible for the highest number of cancer deaths worldwide. Lung adenocarcinoma (LUAD) and lung squamous cell carcinoma (LUSC) are subtypes of non-small-cell lung cancer which has the highest frequency of lung cancer cases. We aimed to analyze genomic and transcriptomic variations including simple nucleotide variations (SNVs), copy number variations (CNVs) and differential expressed genes (DEGs) in order to find key genes and pathways for diagnostic and prognostic prediction for lung adenocarcinoma and lung squamous cell carcinoma. We performed a univariate Cox model and then lasso-regularized Cox model with leave-one-out cross-validation using The Cancer Genome Atlas (TCGA) gene expression data in tumor samples. We generated 35- and 33-gene signatures for prognostic risk prediction based on the overall survival time of the patients with LUAD and LUSC, respectively. When we clustered patients into high- and low-risk groups, the survival analysis showed highly significant results with high prediction power for both training and test datasets. Then, we characterized the differences including significant SNVs, CNVs, DEGs, active subnetworks, and the pathways. We described the results for the risk groups and cancer subtypes separately to identify specific genomic alterations between both high-risk groups and cancer subtypes. Both LUAD and LUSC high-risk groups have more downregulated immune pathways and upregulated metabolic pathways. On the other hand, low-risk groups have both up- and downregulated genes on cancer-related pathways. Both LUAD and LUSC have important gene alterations such as CDKN2A and CDKN2B deletions with different frequencies. SOX2 amplification occurs in LUSC and PSMD4 amplification in LUAD. EGFR and KRAS mutations are mutually exclusive in LUAD samples. EGFR, MGA, SMARCA4, ATM, RBM10, and KDM5C genes are mutated only in LUAD but not in LUSC. CDKN2A, PTEN, and HRAS genes are mutated only in LUSC samples. The low-risk groups of both LUAD and LUSC tend to have a higher number of SNVs, CNVs, and DEGs. The signature genes and altered genes have the potential to be used as diagnostic and prognostic biomarkers for personalized oncology.

## 1. Introduction

Lung cancer is the second most frequently diagnosed cancer type and the leading cause of cancer-related mortality worldwide [1]. Lung cancer treatments used in the clinic are surgery, radiotherapy, chemotherapy, targeted therapy, and emerging immunotherapy. The clinical treatment decisions are made based on tumor stage, histology, genetic alterations of a few driver oncogenes for targeted therapies, and patient’s condition [2]. However, most of the patients are diagnosed at an advanced and metastatic stage, with high mortality and poor benefit from therapies [3]. Although the targeted therapeutics and immunotherapeutics including immune-checkpoint inhibitors are introduced for patients at an advanced stage, these options are beneficial only for limited subsets of patients and these patients still can develop resistance [4]. Therefore, the majority of patients with advanced-stage lung cancer die within 5 years of diagnosis [5].

Histologically there are four major types of lung cancer, including small-cell carcinoma (SCLC), and adenocarcinoma, squamous cell carcinoma, large cell carcinoma as grouped non-small-cell carcinoma (NSCLC). Lung adenocarcinoma (LUAD) and lung squamous cell carcinoma (LUSC) account for 50% and 23% of all lung cancers, respectively [6]. Lung cancer is both histologically and molecularly heterogeneous disease and characterizing the genomics and transcriptomics of its nature is very important for effective therapies. Lung cancer has many subtypes with distinct genetic characteristics, resulting in intra-tumoral heterogeneity [7]. 

The Cancer Genome Atlas (TCGA) database serves different types of data such as transcriptome profiling, simple nucleotide variation, copy number variation, DNA methylation, clinical and biospecimen data of 84,392 cancer patients with 68 primary sites [8]. The Cancer Genome Atlas Research Network reported molecular profiling of 230 lung adenocarcinoma samples using mRNA, microRNA and DNA sequencing integrated with copy number, methylation and proteomic analyses. They identified 18 significantly mutated genes, including TP53, KRAS which is mutually exclusive with EGFR, BRAF, PIK3CA, MET, STK11, KEAP1, NF1, RB1, CDKN2A, GTPase gene RIT1, including activating mutations and MGA including loss-of-function mutations. DNA and mRNA sequence from the same tumor highlighted splicing alterations including exon 14 skipping in MET mRNA in 4% of cases. They also showed DNA hyper-methylation of several key genes: CDKN2A, GATA2, GATA4, GATA5, HIC1, HOXA9, HOXD13, RASSF1, SFRP1, SOX17, WIF1, and MYC over-expression was significantly associated with the hyper-methylation phenotype as well [9].

The Cancer Genome Atlas Research Network also profiled 178 lung squamous cell carcinomas and detected mutations in 11 genes, including mutations in TP53 (81%), CDKN2A, PTEN, PIK3CA, KEAP1, MLL2, HLA-A, NFE2L2, RB1, NOTCH1 including truncating mutations and loss-of-function mutations in the HLA-A class I major histocompatibility gene. They identified altered pathways such as NFE2L2 and KEAP1 in 34%, squamous differentiation genes in 44%, PI3K pathway genes in 47%, and CDKN2A and RB1 in 72% of tumors. CNV analysis revealed the amplification of NFE2L2, MYC, CDK6, MDM2, BCL2L1 and EYS, and deletions of FOXP1, PTEN and NF1 genes with previously identified CNV genes, SOX2, PDGFRA, KIT, EGFR, FGFR1, WHSC1L1, CCND1, and CDKN2A. They identified overexpression and amplification of SOX2 and TP63, loss-of-function mutations in NOTCH1, NOTCH2 and ASCL4 and focal deletions in FOXP1 which have known roles in squamous cell differentiation. CDKN2A is downregulated in over 70% of samples through epigenetic silencing by methylation (21%), inactivating mutation (18%), exon 1β skipping (4%), or homozygous deletion (29%) [10].

Recently, many studies have been published on gene expression signatures predicting the survival risk of patients with lung adenocarcinoma. These recent studies have been mostly using TCGA data, but their methods generated different gene signatures. Seven-gene expression signature including ASPM, KIF15, NCAPG, FGFR1OP, RAD51AP1, DLGAP5 and ADAM10 genes, was obtained for early stage cases from seven published lung adenocarcinoma cohorts and the signature showed high hazard rations in Cox regression analysis [11]. Shukla et al. developed TCGA RNAseq data-based prognostic signature including four protein-coding genes RHOV, CD109, FRRS1, and the lncRNA gene LINC00941, which showed high hazard ratios for stage I, EGFR wild-type, and EGFR mutant groups [12]. A prognostic signature that was independent of other clinical factors, was developed and validated based on the TCGA data. Patients were grouped into risk groups using signature genes, and patients with high-risk scores tended to have poor survival rate at 1-, 3- and 5-year follow-up. The developed eight-gene signature including TTK, HMMR, ASPM, CDCA8, KIF2C, CCNA2, CCNB2, and MKI67 were highly expressed in A549 and PC-9 cells [13].

Twelve-gene signature (RPL22, VEGFA, G0S2, NES, TNFRSF25, DKFZP586P0123, COL8A2, ZNF3, RIPK5, RNFT2, ARHGEF12 and PTPN20A/B) was established by using published microarray dataset from 129 patients and the signature was independently prognostic for lung squamous carcinoma but not for lung adenocarcinoma [14]. A four-gene clustering model in 14-Genes (DPPA, TTTY16, TRIM58, HKDC1, ZNF589, ALDH7A1, LINC01426, IL19, LOC101928358, TMEM92, HRASLS, JPH1, LOC100288778, GCGR) was established and these genes plays role in positive regulation of ERK1 and ERK2 cascade, angiogenesis, platelet degranulation, cell–matrix adhesion, extracellular matrix organization and macrophage activation [15].

Lu et.al. identified differentially expressed genes between lung adenocarcinoma and lung squamous cell carcinoma by using microarray data from the Gene Expression Omnibus database. They identified 95 upregulated and 241 downregulated DEGs in lung adenocarcinoma samples, and 204 upregulated and 285 downregulated DEGs in lung squamous cell carcinoma samples, compared to the normal lung tissue samples. The genes play role in cell-cycle, DNA replication and mismatch repair. The top five genes from global network, HSP90AA1, BCL2, CDK2, KIT and HDAC2 have differential expression profiles between lung adenocarcinoma and lung squamous cell carcinoma [16]. Recently, Wu et.al. identified diagnostic and prognostic genes for lung adenocarcinoma and squamous cell carcinoma by using weighted gene expression profiles. The five-gene diagnostic signature including KRT5, MUC1, TREM1, C3 and TMPRSS2 and the five-gene prognostic signature including ADH1C, AZGP1, CLU, CDK1 and PEG10 obtained a log-rank P-value of 0.03 and a C-index of 0.622 on the test set [17].

A considerable number of genetic and transcriptomic alterations have been identified in mostly LUAD and poorly in LUSC. Although many gene expression signatures have been identified in LUAD recently, there is less work on LUSC expression signatures. Additionally, the molecular differences between risk groups of LUAD and LUSC have not yet been systematically described. In this study, we aimed to identify the genomic and transcriptomic differences between risk groups of lung adenocarcinoma and lung squamous cell carcinoma. We performed a univariate Cox model and then Lasso-Regularized Cox Model with Leave-One-Out Cross-Validation (LOOCV) by using TCGA gene expression data in tumor samples, and identified best gene signatures to cluster patients into low- and high-risk groups. We generated 35- and 33-gene signatures for prognostic risk prediction based on the overall survival time of the patients with LUAD and LUSC. When we clustered patients into high- and low-risk groups, the survival analysis showed highly significant results for both training and test datasets. Then, we characterized the differences including significant SNVs, CNVs, DEGs and active subnetwork DEGs between risk groups in LUAD and LUSC.

## 2. Materials and Methods

### 2.1. Data

Simple Nucleotide Variation (SNV), Transcriptome Profiling, Copy Number Variation (CNV) and Clinical data of patients who have all of these data types in LUAD and LUSC projects, was downloaded separately using *TCGAbiolinks* R package [18]. Using the same package and the reference of hg38; Simple Nucleotide Variations (SNVs) and Copy Number Variations (CNVs); and transcriptomic variations were processed to identify the genomic alterations of the LUAD and LUSC patients (Table 1). The method described below can be found as flowchart in Figure S1.

### 2.2. Gene Expression Signature Analysis

Clinical data and Gene Expression Quantification data (HTSeq counts) of patients with unpaired RNAseq data (tumor samples without normal samples) was downloaded from the TCGA database using the *TCGAbiolinks* R package. Raw HTSeq counts of tumor samples were normalized by TMM (trimmed mean of M values) method and Log_2_ transformed after filtering to remove genes that consistently have zero or low counts. Univariate Cox Proportional Hazards Regression analysis was performed using *survival* R package [19] to identify survival-related genes. For these survival-related potential biomarker genes (*p* ≤ 0.05), Lasso-Regularized Cox Model (by using minimum lambda calculated in the model) with Leave-One-Out Cross-Validation (LOOCV) was performed to determine a gene expression signature using *glmnet* R package [20]. Multivariate Cox Regression for the signature genes was performed and the predictive performance of the model was scored using *riskRegression* R package [21]. The risk score of each patient was predicted based on multivariate Cox regression model using the *survival* R package. Patients were clustered into high-risk and the low-risk group based on the best cutoff value for ROC, calculated by *cutoff* R package [22].

For the validation of the gene signature, HTSeq counts belonging to the tumor samples of patients who have paired RNAseq data (tumor samples with the paired adjacent normal samples) were downloaded from the TCGA database, filtered, normalized by TMM method and Log_2_ transformed. Multivariate Cox Regression for the signature genes was performed and the predictive performance of the model was scored. The risk score of every patient in the validation group was predicted based on multivariate Cox regression model and each patient was assigned to the high- or low-risk group using the best cutoff value for ROC. These analyses were performed for LUAD and LUSC patients separately.

### 2.3. Differential Expression Analysis

Gene Expression Quantification data (HTSeq counts) of both the primary tumor (TP) and the paired normal tissue adjacent to the tumor (NT) was downloaded from the TCGA database. Raw HTSeq counts of both tumor and normal samples were normalized by TMM method after filtering to remove genes which have zero or low counts. Differentially expressed (*q* < 0.01) genes were determined using *limma* [23] and *edgeR* [24] R packages by limma-voom method with duplicate-correlation function. HUGO symbols and NCBI Gene identifiers of the differentially expressed genes were downloaded using the *biomaRt* R package. This analysis was performed for high- and low-risk group patients of LUAD and LUSC, separately.

### 2.4. Active Subnetwork Analysis

Active subnetworks of the differentially expressed genes were determined using *DEsubs* R package [25]. *DEsubs* package accepts the differentially expressed genes output of the *limma* package along with their FDR adjusted *p* values (*q* values). *DEsubs* package both computes and plots the active subnetworks. All the plots and computations were generated for the high- and low-risk group patients of the LUAD and LUSC projects, separately.

### 2.5. Copy Number Variation Analysis

The Copy Number Variation data of the primary tumor samples of patients was downloaded using *TCGAbiolinks* package (Masked Copy Number Segment as data type). The chromosomal regions which are significantly aberrant in tumor samples were determined and plotted by *gaia* R package [26]. Gene enrichment from genomic regions which have significant differential copy number was performed using *GenomicRanges* [27] and *biomaRt* R packages. R codes used in this analysis were modified from the codes presented at “TCGA Workflow” article [28]. All the computations and the plots were generated for the high- and low-risk groups of LUAD and LUSC projects, separately.

### 2.6. Simple Nucleotide Variations Analysis

The masked Mutation Annotation Format (maf) files of the TCGA mutect2 pipeline in tumor samples were downloaded to obtain the somatic mutations. The maf files are filtered using the *maftools* [29] to obtain the subset of the mutations corresponding to the patient barcodes. Summary plot and oncoplot were generated to summarize the mutation data using *maftools* R package. Somatic mutations were filtered and assigned to either oncogene (OG) or tumor suppressor gene (TSG) groups along with a significance score (q < 0.05) using the *SomInaClust* R package [30]. *SomInaClust* computes a background mutation value to identify the hot spots using the known set of somatic mutations in “COSMIC” and the “Cancer Gene Census” (v92) datasets of COSMIC database for GRCh38 [31]. SNV analysis was performed for high- and low-risk group patients of LUAD and LUSC projects, separately.

### 2.7. Visualization

Scatter plots showing risk score and survival time of patients were generated by *ggrisk* R package [32] and Kaplan–Meier (KM) survival curves were plotted by *survminer* R package [33] displaying the overall survival difference between the risk groups stratified on the proposed gene signature. ROC curves were plotted for the risk scores based on each gene signature using *survivalROC* R package [34]. Univariate and multivariate Cox regression analyses were performed and forest plots were generated for risk score with clinical variables using *survival* and *forestmodel* [35] R packages. 

Gene and pathway enrichment analyses were performed by *biomaRt* [36] and *clusterProfiler* [37] R packages and plotted by *enrichplot* R package [38]. Heatmap plots were generated using *ComplexHeatmap* R package [39]. Mosaic plots to compare the categorical variables were generated using the *vcd* R package [40,41].

OncoPrint showing CNVs among patient samples was generated using *ComplexHeatmap* R package. OncoPlot for significant mutated genes was drawn using *maftools,* and oncoPrint showing SNVs and CNVs together was generated using *ComplexHeatmap* R package. Circos plot showing all non-synonymous SNVs in original data of risk groups and significant CNVs at genome-scale were generated using *circlize* R package [42].

All possible relations between DEGs; active subnetwork DEGs; CNV genes; SNV genes of LUAD and LUSC risk groups were identified by using *VennDiagram* R package [43].

## 3. Results

### 3.1. Gene Expression Signature Analysis of LUAD and LUSC Patients

In order to identify gene expression prognosis risk model, clinical data and gene expression quantification data of tumor samples of patients with LUAD and h LUSC with unpaired RNAseq data as two separate training groups (Table 1) were downloaded from the TCGA database. A 35-gene expression signature for LUAD and a 33-gene expression signature for LUSC were identified by Lasso-Regularized Cox Model with LOOCV after univariate Cox regression analysis. The risk scores of each patient in training groups and test groups were predicted using signature genes, then patients were clustered into high- and low-risk groups based on the cutoff values.

The genes of the LUAD expression signature model identified are AC005077.4, AC113404.3, ADAMTS15, AL365181.2, ANGPTL4, ASB2, ASCL2, CCDC181, CCL20, CD200R1, CPXM2, DKK1, ENPP5, EPHX1, GNPNAT1, GRIK2, IRX2, LDHA, LDLRAD3, LINC00539, LINC00578, MS4A1, OGFRP1, RAB9B, RGS20, RHOQ, SAMD13, SLC52A1, STAP1, TLE1, U91328.1, WBP2NL, ZNF571-AS1, ZNF682, ZNF835. Twenty-seven of them are protein-coding genes while two of them are long intergenic non-protein coding RNA (LINC00539, LINC00578), one is antisense RNA (ZNF571-AS1), three of them are pseudogenes (AC005077.4, AC113404.3, OGFRP1) and two of them are novel transcripts (AL365181.2, U91328.1) (Table S1). Pathway enrichment analysis by using *clusterProfiler* R package did not give any results for this 35-gene list; therefore, enrichment analysis was performed manually using the online KEGG Mapper tool. The genes play role in metabolic pathways, cancer and immune system-related pathways such as Central carbon metabolism in cancer, Glycolysis, Cholesterol metabolism, Amino sugar and Nucleotide sugar metabolism, HIF-1 signaling pathway, TNF signaling pathway, IL-17 signaling pathway, Chemokine signaling pathway and Wnt signaling pathway (Table S2). Multivariate Cox regression analysis was performed for the signature genes and the predictive performance of the model was scored. The AUC was 0.963 (*p* = 1.1 × 10^−15^) for LUAD training group. The risk score of each patient was predicted and patients were clustered into high- and low-risk groups based on the cutoff value. Low- and high-risk groups have different expression patterns of the signature genes and significantly different survival probabilities (*p* < 0.0001). The prediction power of the risk score is around 0.78 (AUC) for 1, 3, 5 and 8 years for LUAD training group (Figure S2). Risk group clustering is independent from tumor stages because risk groups have also significantly different survival probability for each tumor stage (Figure S3). Vital status is highly correlated with risk groups that high-risk group is positively correlated with death (*p* = 1.5 × 10^−13^), while only tumor stage IA and III are associated with risk groups (Figure S4). The risk score has highly significant prognostic ability (HR:2.59, *p* < 0.001) when multivariate Cox regression analysis was performed with other clinical variables (Figures S5 and S6).

**Figure 1:**
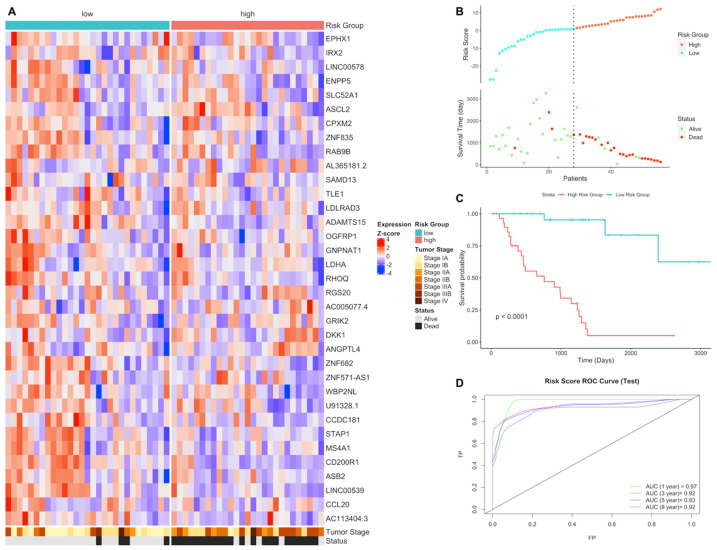
Gene expression signature and risk clustering of LUAD test dataset. Test dataset patients were clustered into high- and low-risk groups based on risk scores of patients calculated by predicting the effect of the signature genes of the signature genes expression on overall survival. (**A**) Expression heatmap of the signature genes in tumor samples of LUAD patients in the test dataset. (**B**) Scatter plot showing risk scores, survival time and separation point of the patients into risk groups. (**C**) KM survival plot showing the overall survival probability between risk groups. (**D**) ROC curve showing prediction power of risk score in the test dataset for 1, 3, 5 and 8 years.

In order to validate the gene expression signature, gene expression quantification data of tumor samples of patients with LUAD who have paired RNAseq data were downloaded from the TCGA database. The risk scores of each patient in test group were predicted using the gene signature lists and patients were clustered into high- and low-risk groups based on the best cutoff values for ROC. Risk groups have differential signature gene expression patterns; high-risk group has lower survival time and higher number of deaths resulting a significantly different survival probability (*p* < 0.0001). The risk score has high prediction powers, 0.97, 0.92, 0.93 and 0.92 (AUC) for 1, 3, 5 and 8 years, respectively, for LUAD test group (Figure 1).

Risk groups have significantly different survival probability for each tumor stage in LUAD test group as well (Figure S7). Vital status is highly correlated with risk groups. The high-risk group is positively correlated with death (*p* = 3.87 × 10^−7^), while only tumor stage I is positively associated with low-risk group (*p* = 0.016) (Figure S8). The risk score has highly significant prognostic ability (HR:2.79, *p* < 0.001) as the result of multivariate Cox regression analysis was performed with other clinical variables (Figure S9).

Expression signature model identified for LUSC includes these genes: AC078883.1, AC096677.1, AC106786.1, ADAMTS17, ALDH7A1, ALK, COL28A1, EDN1, FABP6, HKDC1, IGSF1, ITIH3, JHY, KBTBD11, LINC01426, LINC01748, LPAL2, NOS1, PLAAT1, PNMA8B, RGMA, RPL37P6, S100A5, SLC9A9, SNX32, SRP14-AS1, STK24, UBB, UGGT2, WASH8P, Y_RNA, ZNF160, ZNF703. Twenty-three of them are protein coding genes while two of them are long intergenic non-protein coding RNA (LINC01748, LINC01426), one is antisense RNA (SRP14-AS1), three of them are pseudo-genes (LPAL2, RPL37P6, WASH8P), three of them are novel transcripts (AC106786.1, AC096677.1, AC078883.1) and one is Y RNA (Table S3). They play role in mostly in metabolic pathways, cancer and immunity related pathways such as Arginine and proline metabolism, Glycolysis/Gluconeogenesis, HIF-1 signaling pathway, Non-small-cell lung cancer, PD-L1 expression and PD-1 checkpoint pathway in cancer and TGF-beta signaling pathway (Table S4).

The predictive performance score of the signature model is 80.8 (AUC) (*p* = 1.3 × 10^−6^) in multivariate Cox regression analysis for LUSC training group. The risk score of each patient was predicted and patients were clustered into high- and low-risk groups based on the cutoff value. Low- and high-risk groups have different expression patterns of the signature genes and significant difference of survival probability (*p* < 0.0001). The AUC values showing prediction power of the risk score are 0.76, 0.82, 0.87 and 0.92 for 1, 3, 5 and 8 years, respectively, for LUSC training group (Figure S10). Risk groups have also significantly different survival probability for tumor stages I, II and III (Figure S11). Risk groups are highly correlated with vital status. The high-risk group has highly significant positive correlation with death (*p* = 8.5 × 10^−15^), while low-risk group is negatively correlated. Tumor stages did not show any association with risk groups (Figure S12). The risk score has highly significant prognostic ability (HR:2.85, *p* < 0.001) when multivariate Cox regression analysis was performed with other clinical variables (Figure S13).

**Figure 2:**
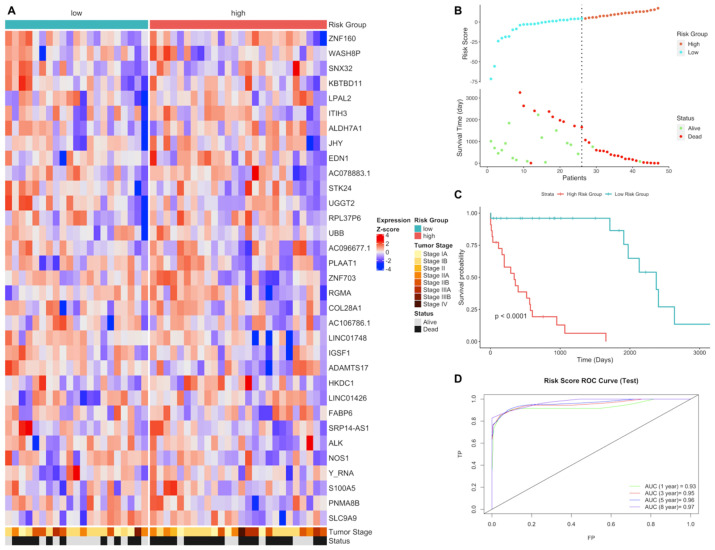
Gene expression signature and risk clustering of LUSC test dataset. Test dataset patients were clustered into high- and low-risk groups based on risk scores of patients calculated by predicting the effect of the signature genes’ expression on overall survival. (**A**) Expression heatmap of the signature genes in tumor samples of LUSC patients in the test dataset. (**B**) Scatter plot showing risk scores, survival time and separation point of the patients into risk groups. (**C**) KM survival plot showing the overall survival probability between risk groups. (**D**) ROC curve showing prediction power of risk score in the test dataset for 1, 3, 5, and 8 years.

In order to validate the gene expression signature for LUSC, gene expression quantification data of tumor samples of patients with LUSC who have paired RNAseq data were downloaded. The risk scores of each patient in LUSC test group were predicted using gene signature lists and patients were clustered into high- and low-risk groups based on the best cutoff values for ROC. Risk groups have differential signature gene expression pattern; high-risk group has lower survival time and higher number of deaths. Risk groups have significantly different survival probability (*p* < 0.0001). The risk score has high prediction powers, 0.93, 0.95, 0.96 and 0.97 (AUC) for 1, 3, 5 and 8 years, respectively, for LUSC test group (Figure 2).

Risk groups have also significantly different survival probability for tumor stages in test group (Figure S14). Vital status is not correlated with risk groups of LUSC test group that number of deaths is higher for high-risk group insignificantly (*p* = 0.07). Tumor stages are not associated with risk groups (Figure S15). The risk score has highly significant prognostic ability (HR:2.66, *p* < 0.001) while other clinical variables have no effect on overall survival in multivariate Cox regression analysis (Figure S16).

The expression gene signatures of LUAD and LUSC do not have any common gene, however they share eight common pathways which are mostly metabolic pathways: Central carbon metabolism in cancer, Glycolysis/Gluconeogenesis, HIF-1 signaling pathway, Pyruvate metabolism, PPAR signaling pathway, Amino sugar and nucleotide sugar metabolism, TNF signaling pathway and Pathways of neurodegeneration—multiple diseases.

### 3.2. Differential Expression and Active Subnetwork Analysis of Risk Groups

Gene expression quantification data of both primary tumor and adjacent normal tissues of patients who have paired RNAseq data (test groups) in LUAD and LUSC projects were downloaded from the TCGA database. Differentially expressed (*q* < 0.01) genes (DEGs) were determined in tumor samples according to normal samples for high- and low-risk patient groups in test sets of LUAD and LUSC, separately. Then, active subnetworks of DEGs in tumor samples were determined using the DEGs with their q values.

**Figure 3:**
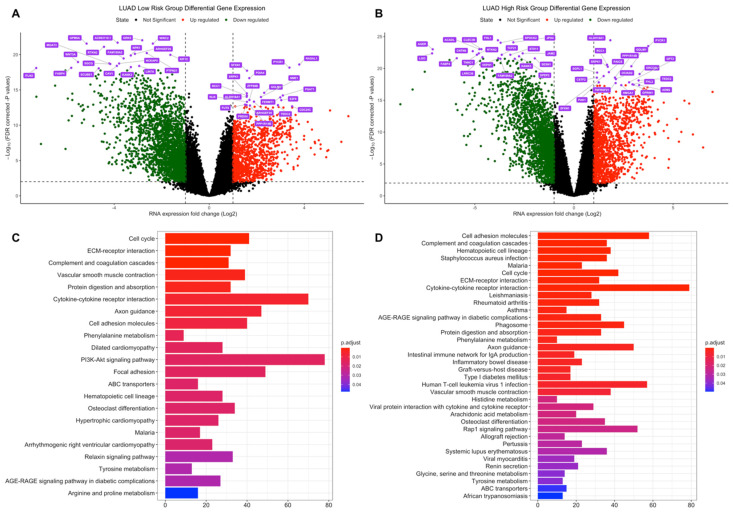
Differential expression analysis of the LUAD risk groups. LUAD test dataset patients were clustered into high- and low-risk groups based on risk scores of patients and differentially expressed genes in tumor samples were determined based on expressions in normal tissues. (**A**) Volcano plot showing differentially expressed genes more than 2-fold (Log_2_ =1) for LUAD low-risk group. The top 20 significant downregulated and upregulated genes are highlighted as purple. FDR corrected p-values threshold is 0.01 (-Log_10_ = 2). Red: Upregulated, Green: Downregulated, Black: Not significant or low than 2-fold. (**B**) Volcano plot showing differentially expressed genes more than two-fold (Log_2_ = 1) for the LUAD high-risk group. The top 20 significant downregulated and upregulated genes are highlighted as purple. FDR corrected *p*-values threshold is 0.01 (-Log_10_ = 2). Red: Upregulated, Green: Downregulated, Black: Not significant or low than 2-fold. (**C**) Dysregulated pathways of differentially expressed genes for LUAD low-risk group. (**D**) Dysregulated pathways of differentially expressed genes for LUAD high-risk group.

In tumor samples of the LUAD low-risk group, the number of the genes which are dysregulated significantly (*q* < 0.01) more than 2-fold is 3615 (2439 down-, 1176 upregulated) while 3610 genes (2239 down-, 1371 upregulated) are dysregulated for the LUAD high-risk group. LUAD low- and high-risk groups have 2745 common differentially expressed genes (Figure S17). The top 20 significant DEGs highlighted as purple at volcano plot in Figure 3A,B are different between LUAD risk groups as dysregulation pattern is different between risk groups albeit the shared 2745 DEGs.

Seven of the signature genes (GNPNAT1, CCDC181, LDHA, ADAMTS15, IRX2, LINC00578, AC005077.4) are dysregulated in both risk groups. ANGPTL4 is upregulated in the high-risk group while MS4A1, GRIK2, and OGFRP1 are upregulated in the low-risk group.

Risk groups of LUAD share dysregulated pathways (Figure 3C,D), highly related to cancer, such as Cell cycle, Biosynthesis of amino acids and Protein digestion and absorption which are upregulated for both risk groups (Figure S18), on the other hand, they also share ECM–receptor interaction, Cell adhesion molecules pathways with immune system-related pathways such as Complement and coagulation cascades and Cytokine-cytokine receptor interaction which are downregulated for both risk groups (Figure S18). However, the high-risk group has more dysregulated immune system-related pathways such as Allograft rejection, Graft-versus-host disease, Inflammatory bowel disease, Intestinal immune network for IgA production, Rheumatoid arthritis, Staphylococcus aureus infection (Figure 3C,D), which are downregulated pathways in LUAD high-risk group (Figure S18).

Active subnetworks of differentially expressed genes in tumor samples of the LUAD risk groups were identified and low-risk group has 191 genes while high-risk group has 168 genes including 112 common genes, which are acting on active subnetworks (Figure S17). 

**Figure 4:**
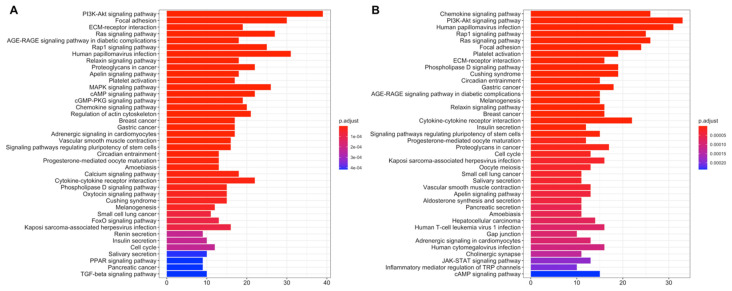
Pathway enrichment of differentially expressed genes at active subnetworks of the LUAD risk groups. Active subnetworks were determined by using differential expression analysis results and pathway enrichment analysis was performed for the genes at subnetworks. (**A**) Pathways of differentially expressed genes in active subnetworks for LUAD low-risk group. (**B**) Pathways of differentially expressed genes in active subnetworks for LUAD high-risk group.

Pathway enrichment of DEGs at active subnetworks shows that the genes playing role in active subnetworks are much more related to cancer pathways such as PI3K-Akt signaling pathway, Ras signaling pathway, Small-cell lung cancer, Breast cancer, Gastric cancer, Proteoglycans in cancer and Rap1 signaling pathway (Figure 4). LUAD risk groups have mostly similar cancer-related active pathways, however only low-risk group has FoxO signaling pathway and TNF signaling pathway while high-risk group has Estrogen signaling pathway, Growth hormone synthesis, secretion, and action with immune system pathways such as Antigen processing and presentation, Intestinal immune network for IgA production and Leukocyte trans-endothelial migration.

**Figure 5:**
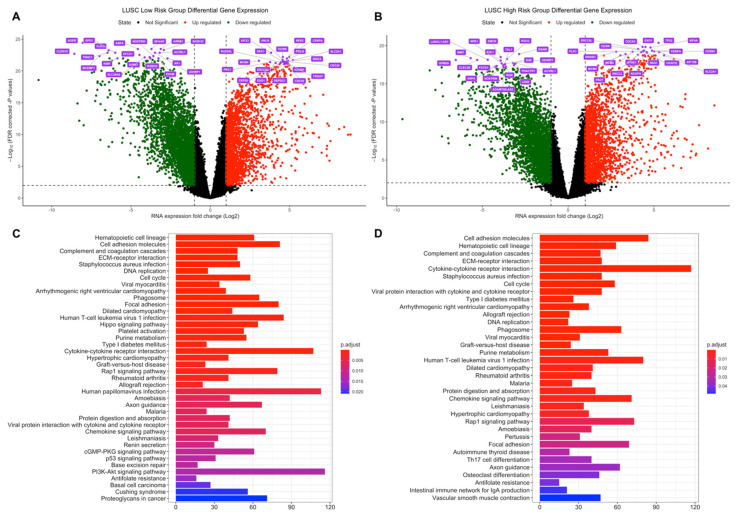
Differential expression analysis of the LUSC risk groups. LUSC test dataset patients were clustered into high- and low-risk groups based on risk scores of patients and differentially expressed genes in tumor samples were determined based on expressions in normal tissues. (**A**) Volcano plot showing differentially expressed genes more than 2-fold (Log_2_ = 1) for LUSC low-risk group. The top 20 significant downregulated and upregulated genes are highlighted as purple. FDR corrected p-values threshold is 0.01 (-Log_10_ = 2). Red: Upregulated, Green: Downregulated, Black: Not significant or low than 2-fold. (**B**) Volcano plot showing differentially expressed genes more than two-fold (Log_2_ = 1) for LUSC high-risk group. The top 20 significant downregulated and upregulated genes are highlighted as purple. FDR corrected p-values threshold is 0.01 (-Log_10_ = 2). Red: Upregulated, Green: Downregulated, Black: Not significant or low than 2-fold. (**C**) Dysregulated pathways of differentially expressed genes for LUSC low-risk group. (**D**) Dysregulated pathways of differentially expressed genes for LUSC high-risk group.

The number of dysregulated genes expressed significantly (*q* < 0.01) more than 2-fold in tumor samples of the LUSC low-risk group is 5596 (3394 downregulated, 2202 upregulated) while 5403 genes (3338 downregulated, 2065 upregulated) are dysregulated for LUSC high-risk group. LUSC low- and high-risk groups have 4562 common differentially expressed genes (Figure S17). The top 20 significant DEGs highlighted at volcano plot in Figure 5A,B include common genes and dysregulation pattern is similar between risk groups.

LUSC signature genes have 10 common genes (EDN1, JHY, PLAAT1, HKDC1, ITIH3, KBTBD11, RGMA, ZNF703, S100A5, LPAL2) with DEGs of both risk groups. Three of the signature genes, ADAMTS17, IGSF1, and LINC01426, are upregulated in the low-risk group; others, NOS1 and SRP14-AS1 are downregulated while Y_RNA is upregulated in the high-risk group.

Risk groups of LUSC have common dysregulated pathways (Figure 5C,D), which are highly related to cancer, such as Cell cycle, DNA replication, Base excision repair, p53 signaling pathway which are upregulated at both risk groups (Figure S19), on the other hand, they also share ECM–receptor interaction, Cell adhesion molecules, Focal adhesion pathways with immune system-related pathways such as Chemokine signaling pathway, Complement and coagulation cascades, Cytokine–cytokine receptor interaction, which are downregulated at both risk groups (Figure S19). However, the high-risk group has more upregulated metabolic pathways such as Central carbon metabolism in cancer, Protein digestion and absorption, Alanine, aspartate and glutamate metabolism, Arginine and proline metabolism, Cysteine and methionine metabolism, Glutathione metabolism, Ribosome biogenesis in eukaryotes; and downregulated immune-related pathways such as JAK-STAT signaling pathway, TNF signaling pathway, Primary immunodeficiency, T cell receptor signaling pathway distinctly from low-risk group (Figure S19). LUSC low-risk group has downregulated PI3K-Akt signaling pathway, Phenylalanine metabolism, Tyrosine metabolism, Phospholipase D signaling pathway, Proteoglycans in cancer and Tight junction pathways with upregulated Hippo signaling pathway and Small-cell lung cancer distinctly from high-risk group (Figure S19).

**Figure 6:**
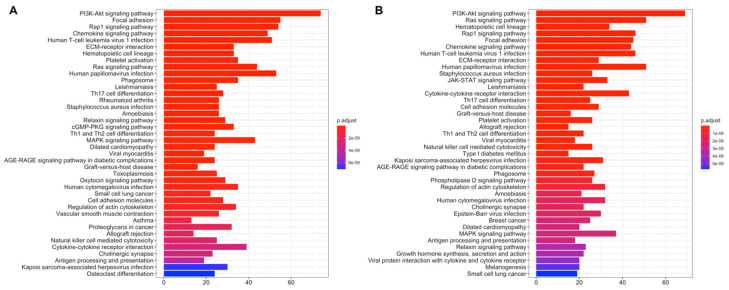
Pathway enrichment of differentially expressed genes at active subnetworks of the LUSC risk groups. Active subnetworks were determined by using differential expression analysis results and pathway enrichment analysis was performed for the genes at subnetworks. (**A**) Active pathways of differentially expressed genes for LUSC low-risk group. (**B**) Active pathways of differentially expressed genes for LUSC high-risk group.

Active subnetworks of differentially expressed genes in tumor samples of the LUSC risk groups has 357 genes for the low-risk group while 350 genes for high-risk group including 245 common genes (Figure S17). Active pathways of the LUSC risk groups, are highly related to cancer pathways such as PI3K-Akt signaling pathway, Ras signaling pathway, Small-cell lung cancer, Proteoglycans in cancer and Rap1 signaling pathway (Figure 6A,B). LUSC risk groups have mostly similar cancer-related active pathways, however only low-risk group has Nucleotide excision repair, Adherens junction and Alpha-Linolenic acid metabolism pathways, while high-risk group has cancer and metabolism-related pathways such as Basal cell carcinoma, Prolactin signaling pathway, Apoptosis, Mitophagy, Choline metabolism in cancer, Insulin signaling pathway, Carbohydrate digestion and absorption, Central carbon metabolism in cancer with immune system-related Measles and Influenza A pathways.

### 3.3. Copy Number Variations Analysis

**Figure 7:**
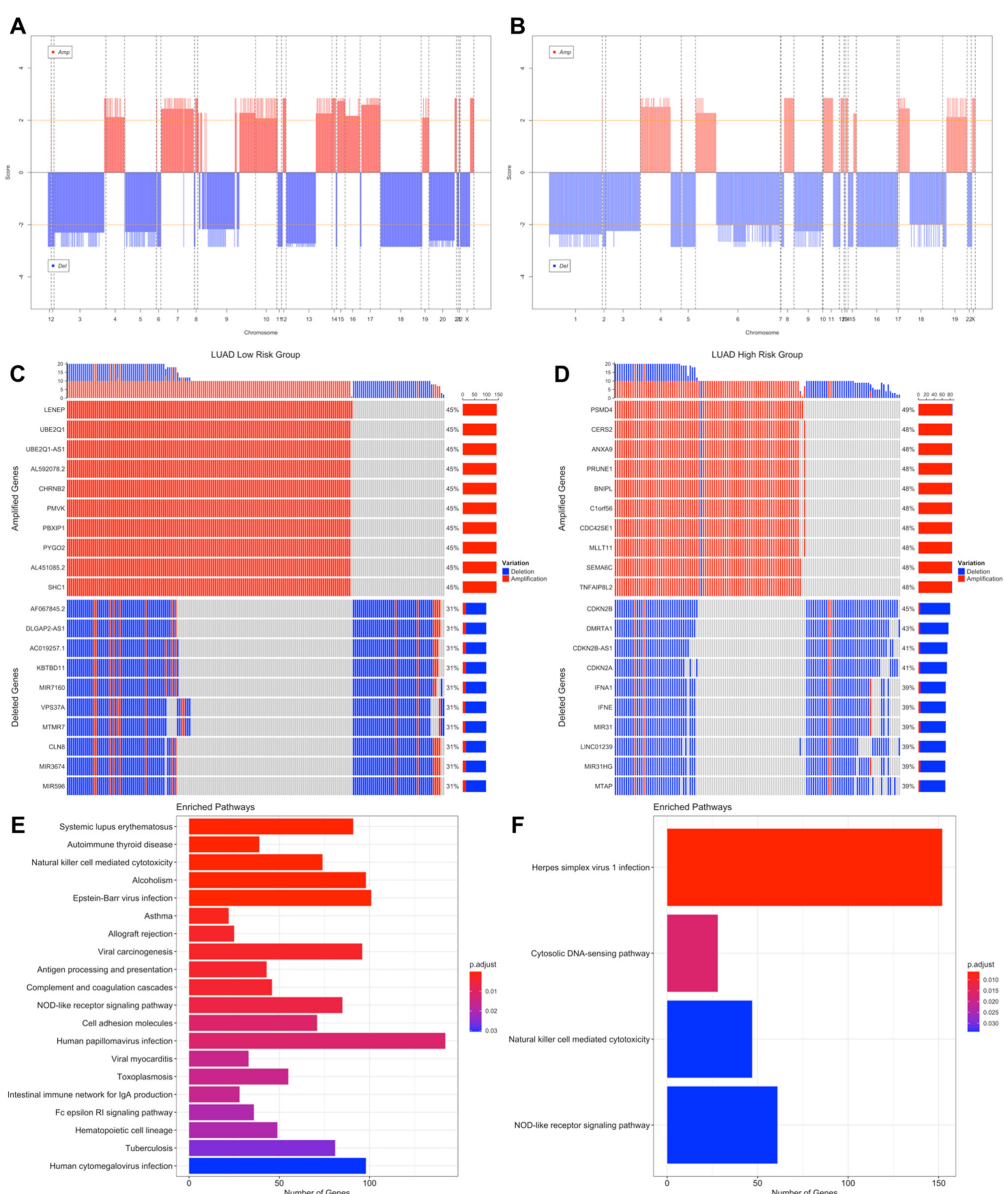
Significant Copy Number Variations (CNVs) of the LUAD risk groups. (**A**) CNV plot at genome scale showing amplified or deleted genomic regions on chromosomes of the LUAD low-risk group. Score: -Log_10_(q value), Horizontal orange line: 0.01 q value threshold. (**B**) CNV plot of the LUAD high-risk group. (**C**) OncoPrint plot showing 25 the highest frequently amplified and deleted genes of the LUAD low-risk group. (**D**) OncoPrint plot showing 25 the highest frequently amplified and deleted genes of the LUAD high-risk group. (**E**) Pathways of CNV genes of the LUAD low-risk group. (**F**) Pathways of CNV genes of the LUAD high-risk group.

The significant aberrant genomic regions in tumor samples of patients were determined and then gene enrichment from genomic regions which have differential copy number was performed. Pathway enrichment analysis of genes which have CNVs was performed and plotted. LUAD low- and high-risk groups have different CNV profiles as seen at CNV plots showing amplified or deleted genomic regions on chromosomes. Chromosomes 1, 6, 7, 10, 13, 16, 17, 28 and 20 have different significant aberrant genomic regions (*q* < 0.01) between risk groups (Figure 7A,B). The highest frequencies of the amplified genes are 45%, 49% and the deleted genes are 31%, 45% in the low- and high-risk groups, respectively. The top 10 the highest frequently amplified or deleted genes in tumor samples of risk groups are different and patients in the same group may have different aberration patterns (Figure 7C,D). The numbers of the deleted genes and the amplified genes are 10,144 and 10,412, respectively, in tumor samples of the LUAD low-risk group. LUAD high-risk group has 5379 deleted and 8442 amplified genes in tumor samples. Risk groups have 4921 deleted and 6559 amplified genes in common (Figure S22).

Pathways of CNV genes are different between LUAD risk groups; mostly immune system pathways such as Allograft rejection, Graft-versus-host disease, Antigen processing and presentation, Complement and coagulation cascades, Inflammatory bowel disease and Viral carcinogenesis pathways have amplified CNVs in the low-risk group (Figure S20) while Herpes simplex virus 1, Cytosolic DNA sensing pathway, Natural killer cell mediated cytotoxicity and Nod-like receptor signaling pathways have deleted CNVs (Figure S20) in the high-risk group (Figure 7). Complement and coagulation cascades pathway has amplified genes in both risk groups while Natural killer cell mediated cytotoxicity and Nod-like receptor signaling pathways have deleted genes in both risk groups (Figure S20). The low-risk group patients have immune system pathways with amplified genes whereas high-risk group patients have immune system pathways with deleted genes. On the other hand, high-risk group has amplified genes in metabolic pathways such as Gastric acid secretion and Insulin secretion (Figure S20).

**Figure 8:**
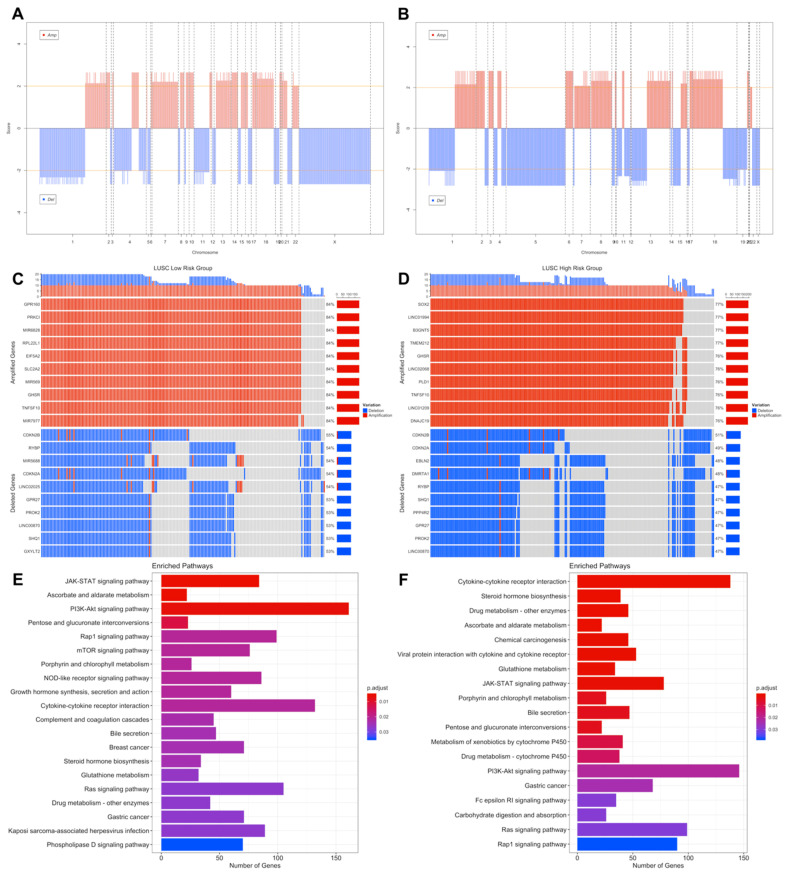
Significant Copy Number Variations (CNVs) of the LUSC risk groups. (**A**) CNV plot at genome-scale showing amplified or deleted genomic regions on chromosomes of the LUSC low-risk group. (**B**) CNV plot of the LUSC high-risk group. (**C**) OncoPrint plot showing 25 the highest frequently amplified and deleted genes of the LUSC low-risk group. (**D**) OncoPrint plot showing 25 the highest frequently amplified and deleted genes of the LUSC high-risk group. (**E**) Pathways of CNV genes of the LUSC low-risk group. (**F**) Pathways of CNV genes of the LUSC high-risk group.

LUSC risk groups have different significant aberrant genomic regions obviously on chromosomes 5, 6, 8 and X (Figure 8A,B). The highest frequencies of amplified genes are 84%, 77% and of the deleted genes are 55%, 51% in the low- and high-risk groups, respectively. LUSC risk groups have higher frequency of amplified genes than deleted genes. Risk groups have common genes from top 25 the highest frequently amplified genes such as SOX2, GHSR, TNFSF10 and miRNAs, miR-7977 and miR-569, with variable frequencies. Risk groups have also common deleted genes such as CDK inhibitors, CDKN2A and CDKN2B, and miR-1284 (Figure 8C,D). LUSC low-risk group has 10,720 deleted and 10,264 amplified genes while LUSC high-risk group has 9477 deleted and 10,250 amplified genes in tumor samples. Risk groups have 7820 deleted and 8659 amplified genes in common (Figure S22).

Pathways of CNV genes highly overlap between LUSC risk groups and they share cancer-related pathways such as PI3K-Akt signaling pathway, JAK-STAT signaling pathway, Ras signaling pathway, Gastric cancer (Figure 8E,F). However, some pathways differ between risk groups, low-risk group has CNVs at mTOR signaling pathway, VEGF signaling pathways and Central carbon metabolism in cancer, while high-risk group has CNVs at Chemical carcinogenesis, Drug metabolism—cytochrome P450, Carbohydrate digestion and absorption pathways (Figure 8E,F). Steroid hormone biosynthesis and Bile secretion pathways have multiple amplified genes while NOD-like receptor signaling pathway has deleted genes, in both risk groups. Only low-risk group has multiple amplified genes at Growth hormone synthesis, secretion and action, and Complement and coagulation cascades pathways. Only high-risk group has amplified genes at Chemical carcinogenesis and Drug metabolism pathways while has deleted genes at Cytokine-cytokine receptor interaction and Fatty acid biosynthesis pathways (Figure S21).

### 3.4. Simple Nucleotide Variations Analysis

Significantly (*q* < 0.05) mutated genes classified as oncogene (OG) or tumor suppressor gene (TSG) based on TSG/OG scores of the genes and the Cancer Gene Census, were identified for LUAD and LUSC risk groups. COSMIC database was used as a reference mutation database for this analysis and Cancer Gene Census data.

**Figure 9:**
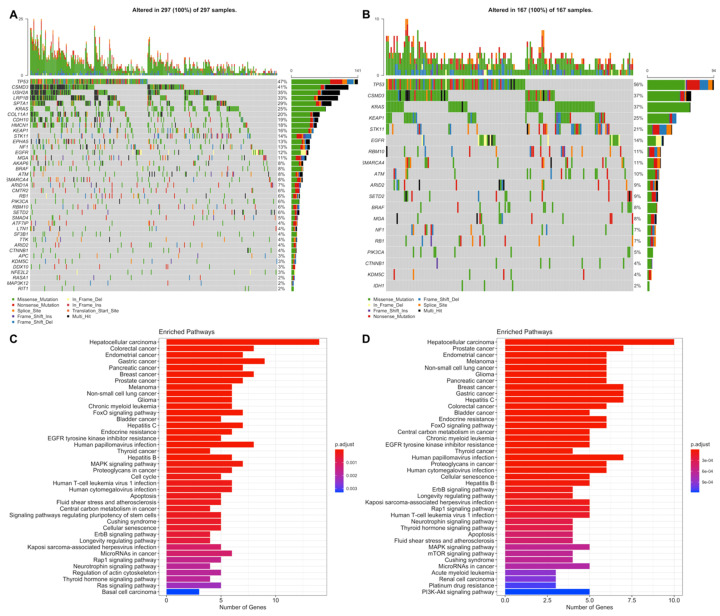
Oncoplot of potential driver genes containing significant SNVs of the LUAD risk groups. (**A**) Oncoplot showing significant SNV genes in tumor samples of the LUAD low-risk group patients. (**B**) Oncoplot showing significant SNV genes in tumor samples of the LUAD high-risk group patients. (**C**) Pathway enrichment of the significant SNV genes of the LUAD low-risk group. (**D**) Pathway enrichment of the significant SNV genes of the LUAD high-risk group.

LUAD low-risk group has 15,376 mutated genes, while LUAD low-risk group has 12,815 mutated genes, 11,516 genes of which are common between LUAD risk groups (Figure S27). LUAD patients have a wide range of mutation numbers changing from 1518/1158 to 10s with median 167 and 172.5 for low- and high-risk groups, respectively. Missense mutation is the highest frequent mutation type, and C > A and C > T substitutions are the most frequent ones for both risk groups. LUAD risk groups have a similar set of mutated genes with varying frequencies. TP53 is the highest frequently mutated gene with 45% and 53% for low- and high-risk groups, and the following ones are MUC16 (39%, 40%) and CSMD3 (38%, 35%) for both groups (Figure S23). SomInaClust analysis was performed to determine driver genes, and 39 genes and 19 genes are strong candidate driver genes for the low-risk group and high-risk group, respectively (Tables S5 and S6). Interestingly, LUAD risk groups share 18 of these driver genes (Figure S27). SomInaClust calculates TSG and OG scores based on background mutation rate and hot spots, then classifies the genes based on TSG/OG scores and cancer gene census data (Figure S25). The driver genes determined in LUAD low-risk group are KRAS, TP53, EGFR, BRAF, STK11, MGA, NF1, RB1, PIK3CA, ATM, RBM10, SETD2, ARID1A, CTNNB1, CMTR2, SF3B1, CSMD3, ATF7IP, KEAP1, HMCN1, EPHA5, ARID2, TTK, SMAD4, KDM5C, SMARCA4, APC, NFE2L2, RIT1, DDX10, LTN1, CDH10, SPTA1, LRP1B, COL11A1, MAP3K12, USH2A, AKAP6 and RASA1. The driver genes determined in LUAD high-risk group are KRAS, TP53, STK11, EGFR, BRAF, RBM10, PIK3CA, SETD2, ARID2, NF1, RB1, MGA, KEAP1, CSMD3, SMARCA4, CTNNB1, KDM5C, IDH1 and ATM (Figure S25; Tables S5 and S6). TP53 and CSMD3 genes are the most frequently mutated genes with 47%, 56% and 41%, 37% frequencies, respectively for low- and high-risk groups (Figure 9A,B). More than half of the genes are mutated in less than 12% of patients. For common genes, LUAD high-risk group has mostly higher frequencies. TP53 has differential mutation types, while KRAS has mostly missense mutations. CSMD3 has more multi-hits (multiple mutations in one patient) in the low-risk group than the high-risk group. EGFR has in frame deletions in both risk groups and other common genes have similar mutation type pattern between risk groups (Figure 9A,B). Pathways of driver mutated genes are highly lung cancer-related pathways such as Non-small-cell lung cancer, EGFR tyrosine kinase inhibitor resistance, Platinum drug resistance, MAPK signaling, mTOR signaling, Ras signaling pathway, PI3K-Akt signaling (Figure 9C,D) and other immunologic and metabolic pathways such as Signaling pathways regulating pluripotency of stem cells, FoxO signaling pathway, Rap1 signaling pathway, Central carbon metabolism in cancer, Proteoglycans in cancer, Human T-cell leukemia virus 1 infection, PD-L1 expression and PD-1 checkpoint pathway in cancer and Natural killer cell mediated cytotoxicity pathways, for both risk groups. Many common pathways are enriched because these mutated driver genes play role in many crucial important pathways. However, Wnt signaling pathway and Hippo signaling pathways are mutated only in the low-risk group, while Gap junction, GnRH signaling pathway, C-type lectin receptor signaling pathway, T cell receptor signaling pathway, HIF-1 signaling pathway, Growth hormone synthesis, secretion and action and AMPK signaling pathways are mutated only in the high-risk group (Figure 9C,D).

**Figure 10:**
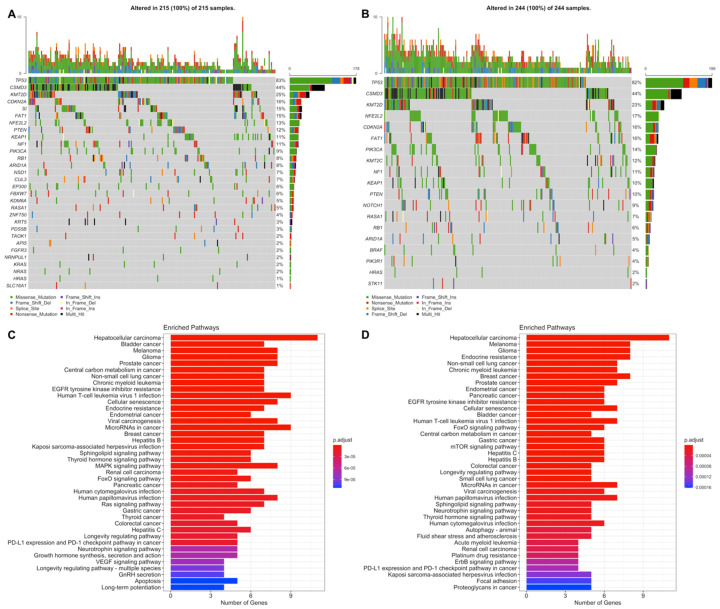
Oncoplot of potential driver genes containing significant SNVs of the LUSC risk groups. (**A**) Oncoplot showing significant SNV genes in tumor samples of the LUSC low-risk group patients. (**B**) Oncoplot showing significant SNV genes in tumor samples of the LUSC high-risk group patients. (**C**) Pathway enrichment of the significant SNV genes of the LUSC low-risk group. (**D**) Pathway enrichment of the significant SNV genes of the LUSC high-risk group.

LUSC low-risk group has 14,038 mutated genes, while LUSC low-risk group has 14,616 mutated genes, and 11,947 genes are common (Figure S27). LUSC patients have a range of mutation numbers from 2300/1488 to 10s with median 201 for low- and high-risk groups, respectively. Missense mutation is the highest frequent mutation type, and C > A and C > T substitutions are the most frequent ones for both risk groups. LUSC risk groups have overlapping list of mutated genes with varying frequencies. TP53 is the highest frequently mutated gene with 80% and 78% for low- and high-risk groups, and the following ones are CSMD3 (42%, 42%) and MUC16 (39%, 40%) for both groups (Figure S24). As candidate driver genes, 30 genes and 19 genes were identified for the low-risk group and the high-risk group, respectively (Tables S7 and S8). LUSC risk groups share 14 of these driver genes (Figure S27). The driver genes determined in LUSC low-risk group are TP53, KMT2D, NFE2L2, PIK3CA, CDKN2A, PTEN, RB1, FAT1, ARID1A, NF1, RASA1, CUL3, KDM6A, NRAS, KRT5, ZNF750, EP300, FGFR3, TAOK1, CSMD3, NSD1, HRAS, SI, PDS5B, KRAS, KEAP1, API5, HNRNPUL1, SLC16A1, FBXW7. The driver genes determined in LUSC high-risk group are TP53, NFE2L2, PIK3CA, KMT2D, FAT1, CDKN2A, RB1, PTEN, NOTCH1, ARID1A, RASA1, NF1, KMT2C, BRAF, PIK3R1, CSMD3, STK11, HRAS, KEAP1 (Figure S26; Tables S7 and S8). TP53 (83%, 82%), CSMD3 (44%, 44%) and KMT2D (25%, 23%) are most frequent mutated genes for low- and high-risk groups (Figure 10A,B). For common genes, risk groups have similar frequencies. TP53 and KMT2D genes have differential mutation types, while CSMD3 has mostly missense and multi-hit mutations. CDKN2A has mostly truncating mutations in both risk groups and other common genes have similar mutation type pattern between risk groups (Figure 10A,B). Pathways of driver mutated genes are highly lung cancer-related pathways such as Non-small-cell lung cancer, EGFR tyrosine kinase inhibitor resistance, Platinum drug resistance, MAPK signaling and Ras signaling (Figure 10C,D) and other immunologic and metabolic pathways such as FoxO signaling pathway, Central carbon metabolism in cancer, Proteoglycans in cancer, Hepatitis B, Hepatitis C, PD-L1 expression and PD-1 checkpoint pathway in cancer for both risk groups. Many common pathways are enriched because these mutated driver genes play role in many crucial important pathways. However, Gap junction and Ubiquitin mediated proteolysis pathways are mutated only in the low-risk group, while HIF-1 signaling and TNF signaling pathways are mutated only in the high-risk group (Figure 10C,D).

**Figure 11:**
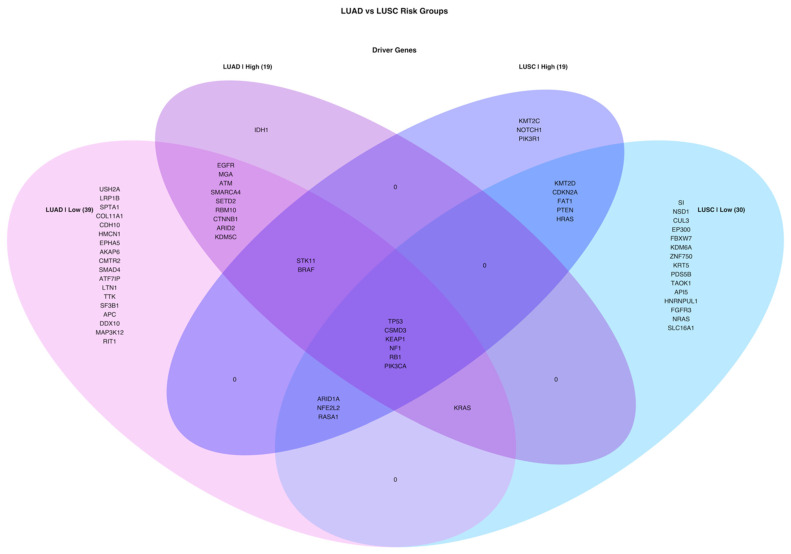
Venn diagram of driver genes containing Simple Nucleotide Variation (SNV) in tumor samples of LUAD and LUSC risk groups.

When venn diagram is drawn by using all driver genes, all cancer and risk groups have TP53, CSMD3, KEAP1, NF1, RB1 and PIK3CA mutations. KRAS, STK11, BRAF, ARID1A, NFE2L2 and RASA1 genes are shared by 3 different groups. LUAD high-risk group has only IDH1 oncogene as different from LUAD low-risk group while LUSC high-risk group has KMT2C, NOTCH1 and PIK3R1 tumor suppressor genes as different from LUSC low-risk group. EGFR, MGA and SMARCA4 are not driver genes in LUSC while CDKN2A, PTEN, HRAS and FAT1 are not driver genes in LUAD groups (Figure 11).

Significant SNVs and CNVs on driver genes are co-displayed as OncoPrint. Although there exist some genes with both SNVs and significant CNVs while others have only SNVs. Moreover, some patients have only SNVs or only CNVs or both for a particular driver gene. 

**Figure 12:**
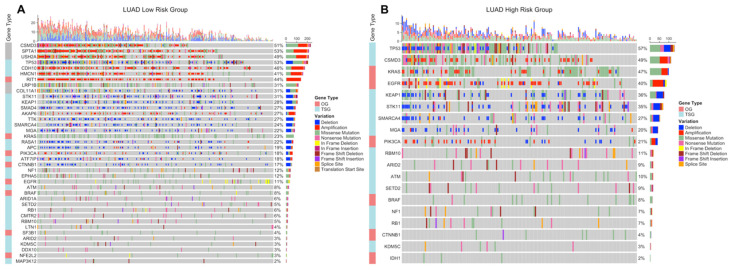
OncoPrint of the driver genes containing significant SNVs and CNVs in LUAD risk groups. Significant SNVs and CNVs are plotted together on potential driver genes in tumor samples of the LUAD risk groups. (**A**) OncoPrint of the driver genes in LUAD low-risk group. (**B**) OncoPrint of the driver genes in LUAD high-risk group.

TP53, STK11, KEAP1, SMARCA4 and MGA genes have deletions while CSMD3 and PIK3CA genes have amplification beside SNVs in both LUAD risk group. KRAS and EGFR genes have amplification in the high-risk group; however, they do not have significant CNVs in the low-risk group. Oncogenes tend to have amplifications while tumor suppressor genes tend to have deletions in both risk groups with exceptions (CSMD3, CDH10, HMCN1, AKAP6 and CTNNB1) (Figure 12).

**Figure 13:**
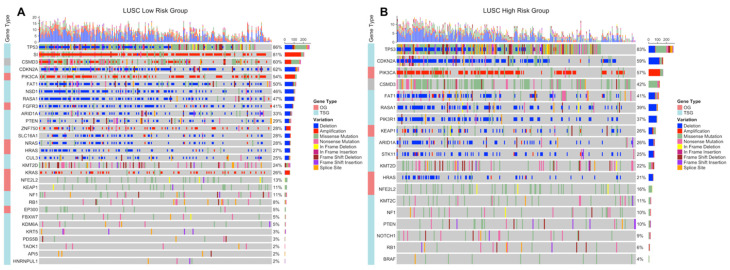
OncoPrint of the driver genes containing significant SNVs and CNVs in LUSC risk groups. Significant SNVs and CNVs are plotted together on potential driver genes in tumor samples of the LUSC risk groups. (**A**) OncoPrint of the driver genes in LUSC low-risk group. (**B**) OncoPrint of the driver genes in LUSC high-risk group.

OncoPrints in Figure 13 show that TP53, CDKN2A, FAT1, RASA1, ARID1A and HRAS genes have deletions while only PIK3CA gene has amplification beside SNVs in both LUSC risk groups. PIK3R1, KEAP1 and STK11 genes have deletions only in the high-risk group while SI, CSMD3, ZNF750, KRAS genes have amplification and NSD1, FGFR3, PTEN, SLC16A1, NRAS and CUL3 have deletion only in the low-risk group. Oncogenes tend to have amplifications while tumor suppressor genes tend to have deletions in both risk groups with exceptions (CSMD3, FGFR3, ZNF750, NRAS, HRAS, KEAP1) (Figure 13).

Circos plots showing all non-synonymous SNVs in original data of risk groups and significant CNVs at genomic scale on chromosomes were drawn to show the genomic alterations between risk groups of LUAD and LUSC.

**Figure 14:**
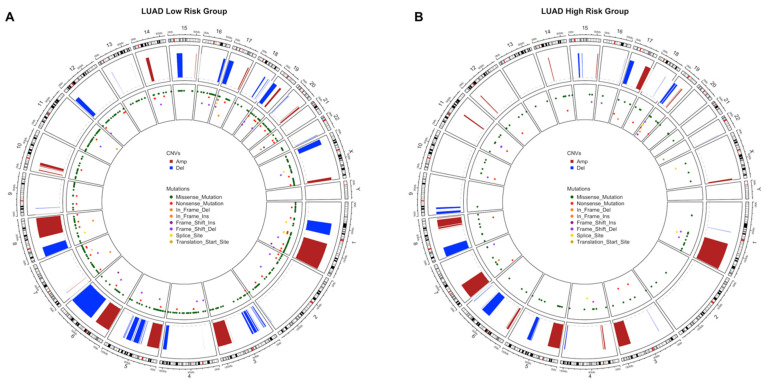
Circos plot of chromosome regions containing all SNVs and CNVs in LUAD risk groups. Significant CNVs (*q* < 0.01) and all SNVs in original data are plotted together on chromosome regions in tumor samples of the LUAD risk groups. (**A**) Circos plot of the LUAD low-risk group. (**B**) Circos plot of the LUAD high-risk group.

LUAD low-risk group has more genome-wide CNVs and SNVs than the high-risk group. The low-risk group has more genomics regions containing missense, nonsense and frame-shift insertions/deletions mutations. Moreover, low-risk group has extra deletions on chromosomes 1, 3, 5, 6, 12, 15 and X with extra amplifications on chromosomes 6, 10, 14, and 20. The high-risk group has extra amplifications on chromosomes 7, 11, 12, and 17. The CNVs of high-risk group are localized mostly on 1, 3, 5, 6, 7, 8 and 17 whereas low-risk group has CNVs on more chromosomes (Figure 14).

**Figure 15:**
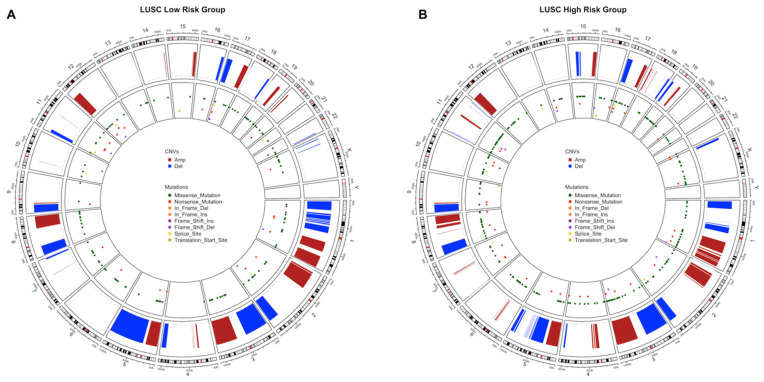
Circos plot of chromosome regions containing all SNVs and CNVs in LUSC risk groups. Significant CNVs (*q* < 0.01) and all SNVs in original data are plotted together on chromosome regions in tumor samples of the LUSC risk groups. (**A**) Circos plot of the LUSC low-risk group. (**B**) Circos plot of the LUSC high-risk group.

LUSC high-risk group has more genomic regions containing missense and nonsense mutations than the low-risk group. However, they have similar amount of CNVs although with different localizations. The high-risk group has extra amplifications on chromosomes 4, 6 and 11; has extra deletions on chromosomes 15, 19 and X. The low-risk group has only extra deletions on chromosomes 1, 5, 6, 11 and 16 (Figure 15).

## 4. Discussion

In order to profile the genetic differences between risk groups of LUAD and LUSC, gene expression signatures were generated and the patients were clustered into low- and high-risk groups and then significant DEGs, DEGs at active subnetworks, CNVs and SNVs were identified in each risk group. The biological alterations for these data types were compared between risk groups and between lung cancer subtypes.

Expression signature for LUAD consists of 35 gene which 27 of are protein-coding genes while two are long intergenic non-protein coding RNA, one is antisense RNA, three are pseudogenes and two are novel transcripts. Many of the coding genes are lung cancer or other cancer types related such as ADAMTS15 [44], ASB2 [45] and EPHX1 [46] with potential tumor suppressor roles; ANGPTL4 [47], ASCL2 [48], CCL20 [49], DKK1 [50], GRIK2 [51], LDHA [52], RGS20 [53], RHOQ [54], TLE1 [55] and WBP2 [56] with potential oncogenic roles; and CD200 [57], CD200R1 [57], CCDC181 [58], GNPNAT1 [59], IRX2 [60], LDLRAD3 [61], STAP1 [62], LINC00578 [63] with prognostic potential. Moreover, MS4A1 is dysregulated in asbestos-related lung squamous carcinoma [64], RAB9B is a target of miR-15/16 which are highly related to lung cancer [65], LINC00539 is related to tumor immune response [66] while long non-coding RNA, OGFRP1, regulates non-small-cell lung cancer progression [67]. The remaining signature genes, CPXM2, ENPP5, SAMD13, SLC52A1, ZNF682, ZNF835, ZNF571-AS1 and U91328.1, have not been related to carcinoma, yet. However, they showed highly prognostic power through risk score to distinguish low- and high-risk of overall survival in LUAD.

LUSC gene expression signature including 33 genes of which ALDH7A1 [68], ALK [69], EDN1 [70], FABP6 [71], HKDC1 [72], IGSF1 [73], KBTBD11 [74], NOS1 [75], SLC9A9 [76], STK24 [77], UBB [78], ZNF703 [79] have been shown with oncogenic relations while RGMA [80] is candidate tumor suppressors. ITIH3 [81] and S100A5 [82] has been related to prognostic biomarker potentials. Other cancer-related genes are ADAMTS17 [83], LINC01748 [84], LPAL2 [85], SRP14-AS1 [86] and WASH8P [87]. Long intergenic non-protein coding RNA, LINC01426, promotes cancer progression via AZGP1 and predicts poor prognosis in patients with LUAD [88]. COL28A1 has prognostic values in glioblastoma [89]. Many of the genes such as JHY, PLAAT1, PNMA8B, RPL37P6, SNX32, UGGT2 and Y_RNA have not been related to any cancer, yet.

Gene expression signatures of LUAD and LUSC share eight pathways which are mostly metabolic pathways. LUAD signature plays role in immune-related pathways as different from those in LUSC. However, pathway enrichment shows us that risk prediction works on metabolic pathways, therefore if we put a name to important mutations as driver mutations, in this case we can say that reprogramming of energy metabolism is the alternative fuel of the cancer [90,91,92]. The differential expression on them with immune system effect in count can hold the passage of cancer.

High-risk groups of both LUAD and LUSC have more immune pathways including downregulated genes and metabolic pathways including upregulated genes. On the other hand, low-risk groups have both upregulated and downregulated genes on cancer-related pathways. Although LUAD and LUSC seem to have similar characteristics of risk groups, close signature gene pathways and similar differential expression pathways sharing 2106 DEGs in total, they are displayed separately in PCA, especially at analysis of test groups.

At CNV level both risk groups and cancer subtypes have huge number of genes with amplifications or deletions which can cause genomic instability and uncontrolled regulation. Both LUAD and LUSC risk groups have important gene alterations such as CDKN2A and CDKN2B deletions which are associated with NSCLC [93] and promotes KRAS and EGFR mutant tumorigenesis [94,95] while SOX2 oncogene amplification in LUSC which is a common event in squamous cell carcinomas [96,97] and amplification of PSMD4 in LUAD, with oncogenic roles in breast, hepatocellular, colorectal and prostate cancer cells [98,99,100,101]. CNVs also play role in metabolic and immune-related pathways which can differ between risk groups and cancer subtypes. If we look from a higher perspective, the LUAD low-risk group has much more CNVs and SNVs on its genome than the high-risk group. On the other hand, the LUSC high-risk group has more SNVs than the low-risk group while CNVs do not vary too much.

SNV analysis gives similar results with literature for example EGFR and KRAS mutations are mutually exclusive in LUAD samples that is confirmed again [9]. Additionally, EGFR [102], MGA [103], SMARCA4 [104], ATM [105], RBM10 [106] and KDM5C [107] which are lung cancer related genes are mutated only in LUAD but not in LUSC. On the other hand, CDKN2A [108], PTEN [109] and HRAS [110] genes are mutated only in LUSC. In general, low-risk groups have more mutated genes for both LUAD and LUSC samples. When SNV and CNV genes are plotted together, it can be seen that LUAD high-risk group has obvious oncogene amplifications and tumor suppressor deletions, while LUAD low-risk group has both tumor suppressor deletions and tumor suppressor amplifications with a few oncogene amplifications. This SNV and copy number differential pattern can cause differential gene expression profiles and characteristics of tumor. LUSC patients have mostly deletions on driver genes with only PIK3CA [111] and KRAS [111] oncogene amplifications. Both LUSC risk groups have obvious TP53 [111] and CDKN2A tumor suppressor gene deletions, but amplification of CSMD3, which has differential roles in lung cancer [112,113], does not occur in LUSC high-risk group. Again, only these driver genes which have differential alterations and frequencies can create the risk difference based on gene expression levels.

## 5. Conclusions

This study has been performed to profile the genomic and transcriptomic differences not only between LUAD and LUSC but also between risk groups to understand the driving differences between them. Treatment options can vary between cancer subtypes and risk groups because of differential targetable mutation patterns. Nowadays, many groups and government institutions are working on the integration of the drug bioactivity and molecular data to investigate more effective molecularly targeting therapeutics for individual patients for the personalized therapy.

## Figures and Tables

**Table 1:** Summary of clinical variables of train and test group of patients with LUAD and LUSC analyzed in the study.

	LUAD	LUSC		
Category	Train Group (n: 436)	Test Group (n: 56)	Train Group (n: 431)	Test Group (n: 47)
Age at diagnosis (median; range)	66; 33–88	66.5; 42–86	68; 39–90	69; 45–85
Gender				
Female	232	33	112	14
Male	204	23	319	33
Tumor stage				
I	241	28	211	25
II	106	13	138	16
III	68	13	76	5
IV	23	2	6	1
Vital status				
Alive	284	30	275	18
Dead	152	26	156	29
Smoked years (median; range)	33; 2–61	31.5; 4–64	40; 8–62	40; 10–60
Smoked packs per year (median; range)	40; 0.15–154	48; 5–94.5	50; 1–240	50; 2–157.5

## Data Availability

The datasets supporting the conclusions of this article are publicly available and can be downloaded from TCGA data portal (https://portal.gdc.cancer.gov) or by using *TCGAbiolinks* R package [18]. The R code used in this study is available upon request.

## Supplementary Materials

The supplementary data are available online at https://www.mdpi.com/2075-4426/11/2/154/s1; Figure S1: Flowchart of method and used R packages in this study. The other R packages not written in this flowchart can be found at Materials and Method part of the article; Figure S2: Gene expression signature and risk clustering of LUAD training dataset; Figure S3: Survival analysis of risk groups clustered by using signature gene expression at different tumor stages in LUAD training dataset; Figure S4: Mosaic plots showing association analysis of categorical variables for LUAD training dataset. Pearson residuals show the positive (blue) or negative (red) association between levels of categories; Figure S5: Multivariate Cox Regression results of clinical variables and risk score in LUAD training dataset. Only risk score has significant result when all clinical variables are included into multivariate analysis; Figure S6: Multivariate Cox Regression results of selected clinical variables (which have significant results in univariate Cox analysis) and risk score in LUAD training dataset. Risk score, t, n, m stages and history of prior malignancy have significant effects on survival. When pathologic tumor stage is used instead of t, n, m stages, only risk score and history of prior malignancy show significant effect on survival; Figure S7: Survival analysis of risk groups clustered by using signature gene expression at different tumor stages in LUAD test dataset; Figure S8: Mosaic plots showing association analysis of categorical variables for LUAD test dataset; Figure S9: Multivariate Cox Regression results of selected clinical variables (which have significant results in univariate Cox analysis) and risk score in LUAD test dataset. Risk score and n stages have significant effect on survival. When pathologic tumor stage is used instead of t, n, m stages, only risk score shows significant effect on survival; Figure S10: Gene expression signature and risk clustering of LUSC training dataset; Figure S11: Survival analysis of risk groups clustered by using signature gene expression at different tumor stages in LUSC training dataset; Figure S12: Mosaic plots showing association analysis of categorical variables for LUSC training dataset. Pearson residuals show the positive (blue) or negative (red) association between levels of categories; Figure S13: Multivariate Cox Regression results of selected clinical variables (which have significant results in univariate Cox analysis) and risk score in LUSC training dataset. Risk score, tissue or organ of origin, t and n stages and history of prior malignancy have significant effects on survival. When pathologic tumor stage is used instead of t, n, m stages, tissue or organ of origin, risk score and history of prior malignancy show significant effect on survival; Figure S14: Survival analysis of risk groups clustered by using signature gene expression at different tumor stages in LUSC test dataset; Figure S15: Mosaic plots showing association analysis of categorical variables for LUSC test dataset. Pearson residuals show the positive (blue) or negative (red) association between levels of categories; Figure S16: Multivariate Cox Regression results of selected clinical variables (which have significant results in univariate Cox analysis) and risk score in LUSC test dataset. Only risk score has significant effect on survival either t, n, m stages or pathologic tumor stage is used instead of t, n, m stages; Figure S17: Venn diagram of differentially expressed genes in tumor samples of risk groups for LUAD and LUSC test groups; Figure S18: Pathway enrichment of DEGs of LUAD risk groups; Figure S19: Pathway enrichment of DEGs of LUSC risk groups; Figure S20: Pathway enrichment of CNV genes of LUAD risk groups; Figure S21: Pathway enrichment of CNV genes of LUSC risk groups; Figure S22: Venn diagram of genes which have significant copy number alterations in tumor samples of LUAD and LUSC risk groups; Figure S23: Summary of SNVs in LUAD risk groups; Figure S24: Summary of SNVs in LUSC risk groups; Figure S25: SomInaClust result of potential driver genes containing significant SNVs in LUAD risk groups. SomInaClust calculates oncogene (OG) score and tumor suppressor gene (TSG) score for each significant gene and classifies the gene according to the score threshold (20) and reference database; Figure S26: SomInaClust result of potential driver genes containing significant SNVs in LUSC risk groups. SomInaClust calculates oncogene (OG) score and tumor suppressor gene (TSG) score for each significant gene and classifies the gene according to the score threshold (20) and reference database; Figure S27: Venn diagram of all genes and potential driver genes containing SNVs of LUAD and LUSC risk groups, Table S1: Gene list of expression signature in LUAD. Ensemble Gene IDs were used in signature analysis and then enriched by using BioMart database; Table S2: KEGG pathway enrichment of expression signature gene list in LUAD by using KEGG Mapper tool; Table S3: Gene list of expression signature in LUSC. Ensemble Gene IDs were used in signature analysis and then enriched by using BioMart database; Table S4: KEGG pathway enrichment of expression signature gene list in LUSC by using *clusterProfiler* R package; Table S5: SomInaClust result of SNV data in tumor samples of LUAD low-risk group; Table S6: SomInaClust result of SNV data in tumor samples of LUAD high-risk group; Table S7: SomInaClust result of SNV data in tumor samples of LUSC low-risk group; Table S8: SomInaClust result of SNV data in tumor samples of LUSC high-risk group.
